# Field-of-view zoom during a single-shot short-axis image for cardiac contraction correction

**DOI:** 10.1186/1532-429X-14-S1-W58

**Published:** 2012-02-01

**Authors:** Peter D Gatehouse, David N Firmin

**Affiliations:** 1 Royal Brompton Hospital, London, UK

## Summary

Evaluate a new method correcting for cardiac contraction during each single-shot image.

## Background

Cardiac motion during a single-shot image causes blurring along the phase-encode axis and dark rim artefact (1). We attempt to correct for myocardial contraction during short-axis single-shot acquisition, to optimise subendocardial myocardium.

## Methods

A myocardial perfusion sequence (balanced SSFP) was modified to vary field-of-view and in-plane offsets during each single-shot rawdata acquisition to “zoom” in [or out] following myocardial contraction [filling]. FOV-zoom varied linearly during raw data acquisition, and for each phase-encode line p scaled the FOV and in-plane offsets by factor Sc(p) = 1 + ((Db - De)/De) x (p-N/2)/N where Db and De were LV short-axis diameter at the begin and end of the single-shot scan, and N was the total phase encode lines (96) for each single-shot. The diameters Db and De were measured using a short-axis cine pre-scan in the same slice. The sequence acquired 300x225mm FOV (2.3x2.3x6mm). For in-vitro tests, a conical cup of fluid was imaged in circular cross-section. The slice was shifted along the cone during acquisition, so that the apparently changing diameter modelled contraction of a short-axis LV. In healthy volunteers without contrast agent, T2-preparation was used for endocardial image contrast (1). Each 200ms perfusion image was acquired during ventricular contraction, starting 80ms after the R-wave, repeated with and without FOV-zoom.

## Results

**Figure 1:**
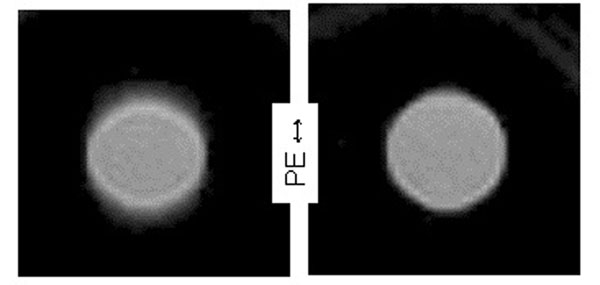
Cup diameter changed 58mm to 39mm during the image. Blurring and band artifact (left) are corrected by FOV-tracking (right) where only Gibbs artifact persists.

**Figure 2:**
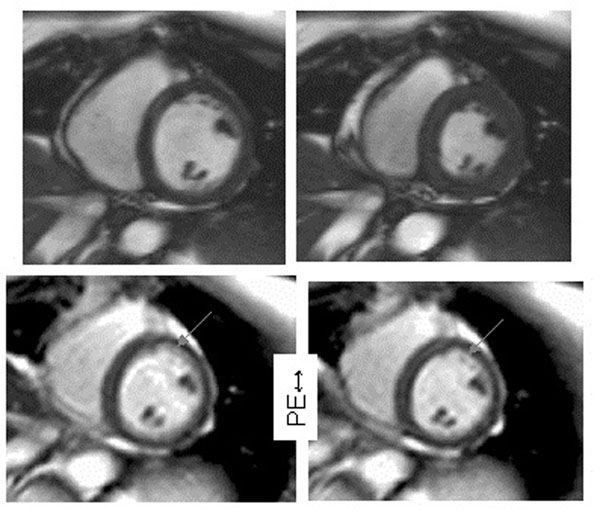
LV diameter changed 46mm (upper left cine frame at 80 ms) to 33mm (upper right at 230ms) during the single-shot image (lower left), showing some endocardial blurring corrected by FOV-tracking (lower right).

Correct operation of the FOV-zoom was confirmed (Figure 1) including in-plane offsets. Gibbs artefact remained unfiltered in all images; the optimum setting of rawdata edge filters during FOV-tracking remains unsolved. Ironically, if FOV-zoom is setup correctly it clarified Gibbs artefact as well as the border. Blurring of the endocardial borders along the phase-encode direction (Figure 2) was reduced by FOV-zoom. The improvement was less than expected given the large change in ventricular diameter (top row) during the image. Although diastolic filling motion is most rapid, the myocardial sectors are more asynchronous even in healthy subjects, and FOV-zoom would be unsuccessful. Even for the more synchronous contraction, the origin of contraction was sometimes midway along the septal endocardial border, and this point must be positioned at the center of the FOV-zoom. Distortion of other tissues by FOV-zoom remained local causing no artefacts at the heart. FOV-zoom might be applied to other long single-shot sequences (e.g. 3D perfusion).

## Conclusions

Some local improvement of subendocardial clarity by FOV-zoom was seen, and ghosting from other tissues was not problematic. However, reliable implementation of this idea remains a challenge.

## Funding

NIHR Cardiovascular Biomedical Research Unit.

